# Dr. Schmidt’s Paper on Bacillus Tuberculosis

**Published:** 1882-12

**Authors:** 


WE publish in this number of the JOURNAL AND EXAMINER, the valuable and instructive paper by Dr. H. D. Schmidt, of New Orleans, to which we have heretofore directed attention by an editorial announcement. The publication of this essay has been awaited with no little impatience, not only in this city, but in several remote parts of the country. The daily press of New Orleans and Chicago, quick to perceive the important character of the claim put forth by the author, attracted public attention to his labors at an early date, and in a way that has given them a wide and deserved publicity. Not a few medical periodicals have given notice of the same announcement.

We can at this point be content to leave our readers to draw their own conclusions as to the essential facts involved; but it is difficult to believe that an issue as important as this will be permitted to rest here. In the next number of the JOURNAL, for example, we expect to publish a supplementary report upon these observations, with an illustrative drawing, by the same author. In this additional paper will be found a record of facts corroborating the views which are set forth in the essay now published, as also a more complete exposition of the method of artificially producing fat-crystals indistinguishable from the so-called bacillus tuberculosis.

We desire to attract to these investigations the attention which they deserve, having in view the end solely sought by the distinguished author, viz., the establishment of assured fact.

SUGGESTIVE.—Farmers in various portions of the world have applied pedigree selection to plants and found that after a few years seventy-two bushels of superior wheat could be produced on one acre. The same is being tried for cotton in India. Medicinal plants, like the rose, the castor oil plant, the cinchona tree, ought to receive particular attention in that direction, selecting from the plants which yield the most efficient oils, alkaloids, etc.

THE Faculty of Medicine of the University of Sidney, include in their curriculum lectures on zoölogy, botany, and comparative anatomy. The course is of five years. Biology is included among the preliminary studies required to enter the medical department of the New Zealand University.—(From the *Australasian Medical Gazette.*) This is another section of the world shut to the American medical practitioner.

